# Ranking the whole MEDLINE database according to a large training set using text indexing

**DOI:** 10.1186/1471-2105-6-75

**Published:** 2005-03-24

**Authors:** Brian P Suomela, Miguel A Andrade

**Affiliations:** 1 Ontario Genomics Innovation Centre, Ottawa Health Research Institute, 501 Smyth Rd, Ottawa, Ontario K1H 8L6, Canada

## Abstract

**Background:**

The MEDLINE database contains over 12 million references to scientific literature, with about 3/4 of recent articles including an abstract of the publication. Retrieval of entries using queries with keywords is useful for human users that need to obtain small selections. However, particular analyses of the literature or database developments may need the complete ranking of all the references in the MEDLINE database as to their relevance to a topic of interest. This report describes a method that does this ranking using the differences in word content between MEDLINE entries related to a topic and the whole of MEDLINE, in a computational time appropriate for an article search query engine.

**Results:**

We tested the capabilities of our system to retrieve MEDLINE references which are relevant to the subject of stem cells. We took advantage of the existing annotation of references with terms from the MeSH hierarchical vocabulary (Medical Subject Headings, developed at the National Library of Medicine). A training set of 81,416 references was constructed by selecting entries annotated with the MeSH term *stem cells *or some child in its sub tree. Frequencies of all nouns, verbs, and adjectives in the training set were computed and the ratios of word frequencies in the training set to those in the entire MEDLINE were used to score references. Self-consistency of the algorithm, benchmarked with a test set containing the training set and an equal number of references randomly selected from MEDLINE was better using nouns (79%) than adjectives (73%) or verbs (70%). The evaluation of the system with 6,923 references not used for training, containing 204 articles relevant to *stem cells *according to a human expert, indicated a recall of 65% for a precision of 65%.

**Conclusion:**

This strategy appears to be useful for predicting the relevance of MEDLINE references to a given concept. The method is simple and can be used with any user-defined training set. Choice of the part of speech of the words used for classification has important effects on performance. Lists of words, scripts, and additional information are available from the web address .

## Background

As the amount of textual information generated by scientific research expands, there is an increasing need for effective literature mining that can help scientists gather relevant knowledge encoded in text documents. The challenge is to develop methods of automated information extraction to support building logical databases and discover new knowledge from online journal collections. A large amount of information for biological research is available in the form of free text such as MEDLINE abstracts. Abstracts are collected and maintained in the MEDLINE database which currently contains references to over 12 million articles dating back to the mid 1960's in domains of molecular biology, biomedicine and medicine, and currently growing by almost half a million articles per year.

MEDLINE articles of interest can be searched for through the PubMed server [1] with queries using a Boolean combination of free text or controlled vocabulary keywords. The usefulness of free text keyword searching will depend on the word content in the title and/or abstract of references of interest. Some interfaces map free text terms to a corresponding Medical Subject Heading (MeSH) [2]. Subject heading (thesaurus, controlled vocabulary) searching can also be a powerful strategy for finding information. Subheadings can help to focus the scope of the search space. This strategy is appropriate for researchers interested in a narrow concept to retrieve a small slice of references for visual inspection. However, there are certain computational analyses of the literature or database developments that would require the ranking of the complete MEDLINE database of references as to their relation to a topic of interest. For example, given any two articles it would be useful to decide which one relates *more *to a topic.

Many machine learning methods have been applied to the problem of document classification [3,4]. Typically such algorithms learn from a set of text that has already been classified (training set) how to classify another set of documents (test set). Naïve Bayes, k-nearest neighbors, decision trees, neural networks, and support vector machines are a few of the most common machine learning algorithms [4]. A key difference between these methods is the way that the documents are represented by the features selected (most often words or phrases from the text) [5]. This results in differences not only in performance but also in the time that is needed to train the method of choice on the test set. Those differences become very significant when the training and the test sets become on the order of thousands. As a result, only naïve Bayesian learning has been applied to the ranking of MEDLINE abstracts (where the test set is on the order of millions of abstracts) and only using training sets of one hundred examples [6]. Just to give an example, a recent survey of these machine learning algorithms on comparatively small sets of text documents required more than five years of CPU time [7]. Applying these methods to classifying thirteen million vectors which are each the width of the number of words used in all the articles in MEDLINE (several hundred thousand even after removal of rare terms and stop words) would certainly be an impossible computational task.

An alternative is given by text indexing based on word frequencies [8]. The titles and abstracts of MEDLINE references contain words that are indicative of specific topics which can be detected by examining how a given word is used more often in references dealing with the topic than in unrelated references. We have previously used this to find keywords in MEDLINE abstracts describing protein families [9] or genetic disease [10], by using the ratio of word usage in a group of pre-selected abstracts with respect to word usage in MEDLINE.

Here we propose to use this approach not just to extract keywords but also to evaluate the entire MEDLINE database with respect to a topic of interest, in a reasonable amount of time such that it can be used in an article search query. The idea is that the learning procedure does not rely on discriminating whole MEDLINE abstracts, but on the words inside, which is much less computationally expensive. This is translated into a dictionary of scored words that can be used later to score any abstract according to the words it contains.

Because the approach is relatively inexpensive, we can evaluate different scoring schemes. We will discuss those and comment on how the performance of the approach is affected by the part of speech (e.g., noun, verb, or adjective) used for the analysis.

## Results

### The training set

The starting point of our algorithm is a set of articles associated (or believed to be associated) with a topic of interest. The system is trained with this set and therefore we define it as the *training set*. To ease evaluation of the method, we chose a subject for which the fraction of articles in the database would be neither too small nor too large of a subset of MEDLINE. In this work we used the topic *stem cells *and we took advantage of the annotation of MEDLINE entries with terms of the MeSH keyword hierarchy to select the training set. For this we obtained by license the complete MEDLINE database (November 2003 release, National Library of Medicine).

The MeSH vocabulary contains 22,568 descriptors, and 139,000 headings called Supplementary Concept Records. An average of 10 MeSH indexing terms are applied to each MEDLINE citation by NLM indexers, who after reading the full text of the article will choose the most specific MeSH heading(s) that describe the concepts discussed. The MeSH indexing terms are organized into concept hierarchies (directed acyclic graphs) that represent *is-a *and *part-whole *relationships [2]. Indexers can also assign subheadings to further describe a particular aspect of a MeSH concept. In addition to assigning MeSH terms that describe the topic of the article, the indexer provides terms that reflect the age group of the population studied, the nature of the studies (e.g. human vs. animal, male vs. female), and the material represented (Publication Types such as *Clinical Trials*, *Editorial*, *Review*). Thus MeSH headings serve as a telegraphic surrogate of the concepts contained in a journal article.

We selected all MEDLINE entries annotated with either the MeSH term *stem cells *or any of its 15 children terms in the MeSH keyword hierarchy (see the list in Table 1). The resulting set contained 81,416 articles with abstracts in MEDLINE (See additional file 1). Entries without abstracts were discarded because our training is based upon the words present in both the abstract and title of the reference. This training set of *stem cell *references represents ~0.5% of MEDLINE.

### Keyword scoring

The property to be analysed is the frequency of certain words in abstracts. Based on our previous experience in the classification of abstracts, we registered the presence of words in the abstract (and title), ignoring the cardinality of words within a single abstract as done in [11], such that a word appearing many times within one abstract would not carry additional weight over a word appearing only once [12]. Additionally, we restricted the analysis to words which commonly convey meaning, that is, nouns, verbs, and adjectives, and not adverbs or conjunctions which would be more appropriate for style studies than for information extraction purposes [13].

Accordingly, we registered the frequencies of 100,196 unique nouns, 20,243 adjectives, and 7,970 verbs in all MEDLINE entries with abstracts (6,803,293 out of a total of 12,330,355 references from the year 1965 until November 2003) that appeared in at least 100 abstracts. Frequencies of 19,117 unique nouns, 6,452 adjectives, and 3,174 verbs were counted in the training set of *stem cell *references. To reduce noise we further filtered the list by considering only words that occurred in more than 100 of the 81,416 entries in the training set (2,256 nouns, 1,193 adjectives, and 748 verbs). Words were always distinguished by their part of speech. For example, of the combined set of 3,449 nouns and adjectives, 104 occurred in the literature both as a noun and as an adjective. Each noun was treated as a separate keyword from its adjective counterpart.

Each word (nouns, adjectives or verbs) occurring in 100 or more of the entries in the training set was scored by the ratio of frequency of occurrence in the training set divided by its frequency of occurrence in all of MEDLINE (see Methods). The top 30 scoring nouns, adjectives, and verbs are listed in Table 2, Table 3, and Table 4, respectively. (The complete lists are given in additional file 2). The set of nouns was much larger than the sets of adjectives or verbs. The top keyword scores were also higher for nouns than for adjectives and verbs.

### Reference scoring

We studied two variables when scoring MEDLINE references on the basis of their word scores: one was the part(s) of speech used, and the other was the number of words used for the score. To make this analysis feasible in terms of computing time, we constructed a set of MEDLINE references with the *training set *and an additional equal number of references with abstracts chosen at random from the rest of MEDLINE, which we will call the *random set*, ideally not related to stem cells. The self-consistency of a given scoring scheme was measured by the fraction of references from the training set that ranked in the top half when the whole set of 162,832 references was scored. The scores for training and random sets are given as supplementary material (additional files 3 and 4, respectively).

We first analyzed the effect of scoring MEDLINE references using the average of all keywords in the abstract and title and compared the results to the average of only the top 5, and the top 10 words with the highest scores, which gave worse results and a small improvement, respectively (data not shown). For the rest of experiments we used the average of all words.

**Figure 1:**
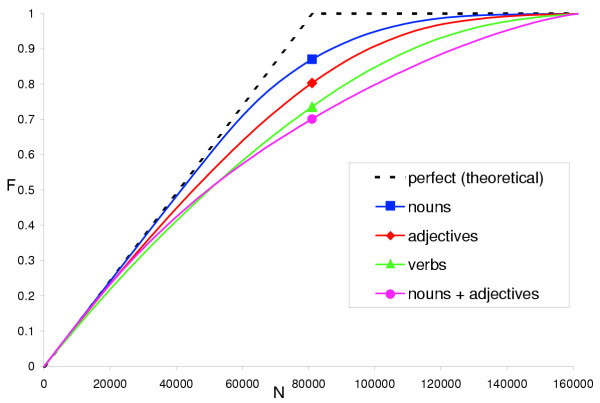
**Self-consistency test of the algorithm**. Fraction of references from the *stem cell *training set (F) retrieved when selecting a number (N) of top-scoring references in a mixed set combining the *training set *and the *random set*. Nouns are better discriminators with F = 0.87 for the top half of the list. F was 0.79 for adjectives, 0.73 for verbs, and 0.70 for nouns plus adjectives. Performance could not be theoretically perfect because there were articles in the training set which were not relevant to *stem cells*, and there were articles in the random set which were relevant to *stem cells*.

We then studied the influence of the part of speech used to score the references. Figure 1 shows the fraction of articles from the training set that was retrieved when selecting a variable range of top-scoring articles. Nouns were better keywords than were adjectives, or verbs. Using both nouns and adjectives as keywords slightly improved retrieval for the top-ranking articles, but weakened prediction of middle and low-ranking stem cell articles. Accordingly, we adopted as a scoring scheme the average over all nouns with scores.

**Figure 2:**
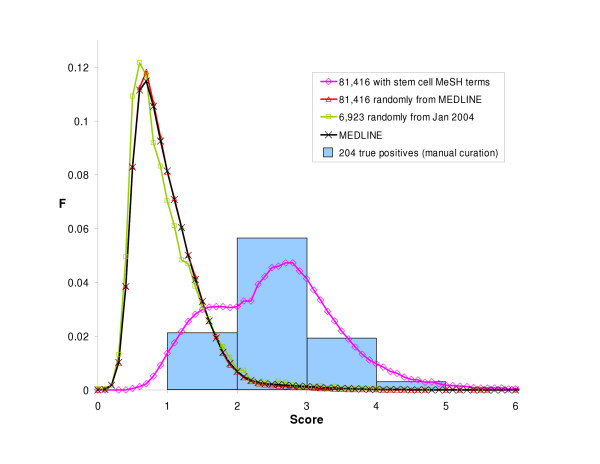
**Distribution of scores in MEDLINE sets**. For each of the sets of MEDLINE references analyzed in this work we plot the distribution of score values (using the average over all nouns). The complete MEDLINE (black line with X's) has a maximum around 0.65. The *training set *composed of 81,416 references annotated with MeSH terms related to *stem cells *(magenta with diamonds) has a maximum at 2.75 and a "hump" at 1.5. This type of distribution is due to the fact that this set includes both references truly related to *stem cells *and others that are not and agree more with the general MEDLINE background distribution of scores. The *random set *of 81,416 references (red with triangles) has, logically, an identical distribution to the whole of MEDLINE. The 6,923 randomly selected MEDLINE references (green with squares) used for the recall and precision test also follow the background distribution. Of those, the 204 references evaluated as *stem cell *related by a human expert (blue bars) had significantly higher scores than the background distribution of MEDLINE.

We computed the scores for all MEDLINE references with abstracts. As expected, the MEDLINE score distribution agreed with the score distribution of the random set and was well below the score distribution of the training set (see Figure 2). However, the considerable overlap between the background and the training set was indicative that neither all references in the training set were dealing with *stem cells *in a strict sense nor all references in the random set were unrelated to *stem cells*.

Close inspection of the top ranking references from the random set revealed that they were also likely to be of interest to anybody wanting to read about stem cells (see Table 5 and Discussion). For these reasons, we measured performance of the method by observing recall and precision in a set evaluated by a human expert and not used to train the algorithm, a typical way to evaluate literature mining algorithms [14].

### Recall and precision of the algorithm

We collected a test set of 6,923 MEDLINE entries randomly chosen from articles published during January 2004, and therefore not included in our training set. Their score distribution was in agreement with the MEDLINE background (Figure 2). According to a human evaluator with expertise in the field of stem-cell biology (MAA) there were 204 articles relevant to the topic of *stem cells *in the set, all with scores clearly above the background.

**Figure 3:**
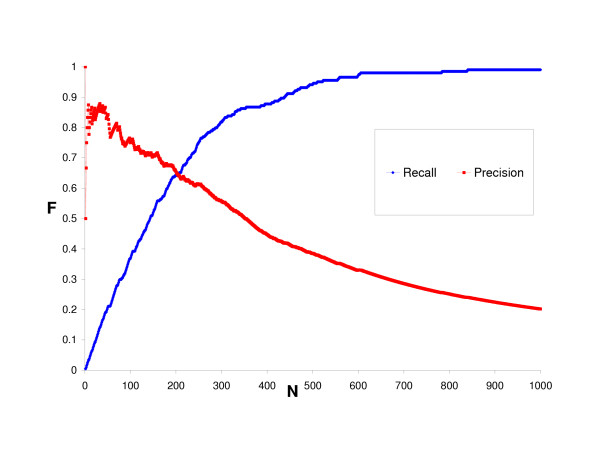
**Recall and precision of the algorithm**. The recall and the precision of the algorithm were checked in a set of 6,923 references not included in the training set. Manual examination of the set resulted in the identification of 204 references (positives) relevant to stem cells. Recall was measured as TP/(TP+FN) and precision as TP/(TP+FP), where TP is true positives, FP is false positives, and FN is false negatives.

Figure 3 displays the recall and precision of the algorithm according to expanding thresholds in the scoring. The selection of the set of the 204 articles with best scores (roughly above a score of 2.14) retrieved 132 true positives and missed 72. For that set, the precision (fraction of selected articles that are true positives) and the recall (fraction of true positives selected) of the algorithm were of 65%.

The first false positive, PMID: 14707522 ranked at position 2, mentions the highly scoring keyword 'fibroblast' (see Table 2) but in the context of an inherited disease (glutaric aciduria type I). Similarly, other articles with high scores that were not considered to be relevant to stem cells by the human expert were usually talking of cells, genes, and proteins relevant to stem cells, but in a context not directly related to stem cell biology, such as cancer or metabolic disease. The worst scoring positive, PMID: 14702195 ranked at position 1910, was a review dealing with the use of neural stem cells for therapy of neurodegenerative diseases. The score was very low because its abstract does not contain any mention to relevant facts about stem cells.

This type of analysis is subjective because it reflects the prejudices of a particular human expert; however, it is indicative of the general agreement between human selection and automated ranking. The complete list of scored abstracts and the results of the human evaluation are available in Additional file 5.

## Discussion

We have introduced a simple strategy to judge the relevance of a text according to a topic of interest based on a training set of text. The method relies on different frequencies of discriminating words between the training set and other non-relevant articles. This algorithm is appropriate for information extraction of molecular biology data from the MEDLINE database of scientific references.

Our analysis of more than six million MEDLINE entries with abstracts indicated that there were 128,409 unique keywords (100,196 nouns, 20,243 adjectives, 7,970 verbs) appearing in at least 100 abstracts. For comparison, the OED, the largest English-language dictionary, contains 290,000 entries with about 616,500 word forms [15]. OED omits many slang words, proper names, scientific and technical terms, and jargon (there are over a million named species of insects). Most estimates of the total vocabulary of English are well over three million words, but only ~200,000 words are still commonly used. An educated person has a vocabulary of ~20,000 words and uses ~2,000 per week through conversation.

To test the system, we constructed a set of references related to the topic of *stem cells *taking those annotated with the corresponding keywords of the MeSH hierarchy (see Methods). This set contained 81,416 MEDLINE references. There were 28,743 unique keywords (19,117 nouns, 6,452 adjectives, 3,174 verbs) extracted from the training set of 81,416 *stem cell *references.

We then focused on words that were used more often in the training set of *stem cell *references than elsewhere. Regarding those words, it was not surprising that a high proportion of the keywords extracted were proper names and scientific jargon. In order to be sure of choosing relevant words (and not those that could be present in the training set by pure chance) we took only those used in more than 100 references in the training set: only 2,256 (12%) of the nouns, 1,193 (18%) of the adjectives, and 748 (24%) of the verbs.

The words were scored by their different usage in stem cell references compared to MEDLINE, and all MEDLINE references with abstracts were ranked by the average of scores of their keywords (see Methods). The best keywords (*mesoderm*, *fibroblast*, *foreskin*, *stem*, *mesenchyme*) were mostly related to sources of stem cells and therefore were identifying relevant references. The worst keywords (*hospital*, *care*, *health*, *practice*, *management*) were totally off-topic and abstracts with many of these generic words would often rank poorly with respect to their relevance to stem cells.

The self-consistency analysis of the algorithm with a set combining the training set with an equally large set of randomly selected references was used to compare the performance of the algorithm for different parts of speech and simple scoring mechanisms. Nouns were found to be superior to verbs and adjectives and the average score of all nouns in the abstract and title was found to be most appropriate. We observed truly *stem cell *related articles in the random set of articles that were not annotated with *stem cells *MeSH terms, and also articles in the abstract set which were not relevant to the subject.

In order to further evaluate the capabilities of the method, we compared the results obtained with those returned by a human expert from a set of 6,923 articles not used for training. A precision of 65% was found for a recall level of 65% in the retrieval of the 204 articles deemed by the human to be relevant.

It would be interesting to see how the algorithm presented here performs when searching for different concepts such as *stem cells*. Evaluation of the self-consistency of the algorithm is relatively simple, so any user can have a good idea of whether there is enough information in the training set to allow distinction from the rest of the database and see how the part of the speech chosen affects performance. However, the least we can do here is to note that the part of speech that gave better performance were nouns. We propose two predictions. Firstly, the optimal part of speech could be related to the part of speech of the topic under consideration; in this case nouns are the best keywords because the topic is an object, *stem cells*; if the topic was a verb, such as *interact *or *phosphorylate*, we expect that a small number of verbs will work better. Our second prediction is that our algorithm will often work better using names as keywords, as it will be easier to discriminate topics composed of nouns or nouns and adjectives than bare adjectives or verbs. This is for the reason that nouns are used to name a person, place, thing, act, or concept, whereas adjectives indicate qualities of the nouns, and verbs tell of doing or being something. Therefore, context is often needed to determine the meaning of adjectives and verbs whereas nouns are relatively context-insensitive, especially in science. Most keywords used in molecular biology [2,16-18], are nouns which are sometimes complemented with an adjective, such as *mitochondrial membrane*.

Ideally biomedical texts should have a lower degree of linguistic variation than other genres [17]. However the naming conventions in biology and biomedicine are highly non-standardized even when it comes to the fundamental concepts. In theory, terms should be mono-referential (one-to-one correspondence between terms and concepts), but in practice we have to deal with *ambiguities *(i.e. homography – the same term corresponds to many concepts) and *variants *(i.e. synonymy – many terms lead to the same concept). One approach to solve the ambiguities of the natural text used in abstracts has been the indexing of the literature in the MEDLINE database by keywords drawn from the MeSH controlled terminology that was originally developed to categorize the citations contained in *Index Medicus*. The annotation of MEDLINE with MeSH terms at the National Library of Medicine helps users to link their search terms to abstracts containing different terms with the same meaning [18].

Annotation of articles with MeSH headings are optionally flagged with subheadings and importance markers (Major / Minor). However some applications might require a fuzzy association to subjects, for example, one reference can be more strongly relevant to *stem cells *than another. This could be important for example when setting up priorities between references. Another reference could be possibly relevant to *stem cells *with a low likelihood. This could matter if a researcher wanted to find out any possible relation of a gene to *stem cells*, even if it is a remote association. The approach presented in this work allows the ranking of any MEDLINE reference with respect to its relevance to a topic.

A different problem with MeSH terms particular to the subject of *stem cells *is that many references were annotated with *stem cells *MeSH terms because of the usage of stem cells as a technique. For example PMID: 15105256 is annotated with the MeSH term *stem cells *because mouse embryonic stem cells were used to raise chimeric mice using a method previously described, yet the major finding of the publication really has nothing to do with *stem cells*. Such an article would likely not be interesting to a researcher working on the biology of stem cells. As discussed previously [19], such information will be contained in the Methods section of the corresponding article and would often be omitted from the abstract. Thus our algorithm defeats this problem by using a different focus to avoid the imprecision caused by trusting MeSH annotations alone.

Regarding the computational time needed by our method, the extraction of specific parts of speech from MEDLINE requires several hours on a reasonably fast machine, but this only has to be done once. Newer entries added to the MEDLINE database can be parsed monthly or more frequently if desired. The main bottleneck is the production of a ranked list of MEDLINE references, which is not a problem if one is interested in only one concept such as *stem cells*. The real limitation arises if one considers using this strategy to the mining of ranked reference lists relating to many concepts. For each concept query, a set of training references must be collected, keyword scoring tables constructed, and all abstracts in MEDLINE must be scored. Providing a real-time interface for arbitrary concept queries is possible but would require some combination of the large storage requirements of pre-processed tables and cluster (or distributed, Beowulf) computing. A more realistic approach would be a local implementation of our approach according to the interests and requirements of individual researchers.

In our implementation, the training set was collected using a selection of MEDLINE references annotated with a subset of terms from the MeSH hierarchy that we considered to be relevant to the subject of *stem cells*. However, there are many other ways of selecting sets of MEDLINE references relevant to topics, such as links from databases like OMIM [10], HSSP [9], or by manual selection. The *garbage in garbage out *principle applies here as in many other applications where the quality of the training set matters, so if the selection is too messy the algorithm might not pick any relevant discriminating keywords.

To make our analysis as impartial and simple as possible, only MeSH terms in the sub tree of *stem cell *were considered, but there are other terms elsewhere in the MeSH hierarchy (e.g. *Embryo Research*) that would also be good indicators that a given article is talking about *stem cells*. It would be feasible to determine the nearest neighbours of an arbitrary MeSH term, and by setting a threshold similarity factor one could include all the MeSH terms within a certain semantic distance of one another in a clustering manner. Surely this would improve the performance of the relevance prediction algorithm. However, considering that we are in a stage of testing and illustrating the method, we employed a simple approach of using a MeSH term and its children in the MeSH hierarchy.

The cosine distance between vectors of word usage can be used to measure distance between MEDLINE abstracts [20]. However, this measure takes into account all the parts of speech, as well as the number of times each word is used in a body of text. The purpose of the cosine measure is to offer an objective distance between entries independent of the user's interest in a particular topic. Therefore our scoring is more appropriate, which is not a distance but rather an absolute value used to derive a ranking upon learning from a training set, a typical strategy in information retrieval [14]. Eventually, the cosine distance might be refined to use only certain parts of speech (such as nouns). We can assume this would give better results when searching MEDLINE neighbours of a given entry in MEDLINE, provided that the user is interested in topics similar to those contained in biological keyword systems.

## Conclusion

This report describes an approach to compute a ranked list of publications according to relevance to a topic of interest, given a training set of MEDLINE references. It is evident that the analysis of the word usage in the abstracts of publications associated with a given concept can be used for literature mining. The strong dependency of the quality of the results with the part of speech used must be taken into consideration. Even if the procedure applied in this work may seem to be too simplistic given the existence of sophisticated methods such as naïve Bayesian classifiers, support vector machines, and neural networks, one should not forget that we are dealing with test sets of millions of abstracts, and training sets of tens of thousands, and that the variation of each single item to be classified is very large because they are composed of some hundred words. In situations like this, sophistication leads very quickly to impossibility of computation and pragmatic approaches are needed. We have produced a method that works and the conclusions obtained regarding the part of speech used may be useful for others working in information extraction from natural language.

## Methods

The databases used were the December 2003 MEDLINE [1] and the 2004 MeSH keyword hierarchy [2]. The *stem cell *training set was selected by taking all references annotated with any MeSH term with a "TreeNumber" identifier of the type A11.872.x.y (for any x and y values).

All titles and abstracts in MEDLINE were processed using the Tree-Tagger part of speech parser [21] to extract separate lists of nouns, adjectives, or verbs, along with their frequency of occurrence. For each keyword found in some training set reference we computed the fraction of references in the training set using the keyword, and the fraction of references in the whole of MEDLINE using the keyword. Each keyword received a score which is the ratio of the frequency of usage in training set over the fraction of usage in the whole of MEDLINE. A score above one indicates that the word was used more often in the training set than in the rest of MEDLINE.

In order to remove irrelevant words associated to the training set by chance one can require that the words appear with a minimum frequency. We chose an absolute number of 100 times in our training set of 81,416 references (~0.1%).

We scored MEDLINE references based on the average score of all keywords in their abstract and title (this is the method used in XplorMed [22]). Words without a score (because they were present less than 100 times in the training set) were not taken into account. For comparison, scores of the top five, or the top ten keywords were also tested. The scoring was performed using nouns, adjectives, verbs, and nouns plus adjectives, as keywords.

## Authors' contributions

BPS designed and tested the scripts that generated and benchmarked the lists of MEDLINE references ranked by their "stemness", and generated the figures of the manuscript. MAA provided advice and guidance, and evaluated the method's precision and recall. Both authors collaborated in the writing of the manuscript.

## Supplementary Material

Additional File 1 ***Stem Cell *references**List in plain text format (stemcellpapers.txt) of 81,416 PubMed Identifiers (PMIDs) linked to abstracts in MEDLINE that have one or more MeSH terms which are members of the set of terms related to *stem cell*. Click here for file

Additional File 2 **Nouns, adjectives, and verb scores**. A zip compressed file (file2.zip) containing three lists in plain text format (sortednounscores.txt, sortedadjectivescores.txt, sortedverbscores.txt) of the computed scores for 2,256 nouns, 1,193 adjectives, and 748 verbs. Click here for file

Additional File 3 ***Stem Cell *MEDLINE reference scores**A zip compressed file (file3.zip) containing lists in plain text format (stemcellpaperscores-adjectives.txt, stemcellpaperscores-nouns.txt, stemcellpaperscores-nounsadjectives.txt, stemcellpaperscores-verbs.txt) of 81,416 PMIDs of stem cell references and their scores according to nouns, adjectives, verbs, and combined nouns/adjectives. Click here for file

Additional File 4 **Subset of MEDLINE reference scores**A zip compressed file (file4.zip) containing lists in plain text format (paperscores-adjectives.txt, paperscores-nouns.txt, paperscores-nounsadjectives.txt, paperscores-verbs.txt) of 81,416 PMIDs of references randomly selected from MEDLINE and their scores according to nouns, adjectives, verbs, and combined nouns/adjectives. Click here for file

Additional File 5 **List of 6,923 scored abstracts**A zip compressed file (file5.zip) containing a table in plain text format with tab separated columns (paperscores-nouns-recent.txt) of 6,923 PMIDs of references not included in the training set with their scores, and a human evaluation of their relevance to the topic of stem cells. Scripts are available on request. TreeTagger is available from [21].
Click here for file

## Figures and Tables

**Table 1:** MeSH keywords that are children of Stem Cell in the MeSH hierarchy

Fibroblasts
Colony-Forming Units Assay
Stem Cell Transplantation
Tumor Stem Cells
Erythroid Progenitor Cells
Myeloid Progenitor Cells
Myocytes, Cardiac
Myocytes, Smooth Muscle
Muscle Cells
Muscle Fibers
Satellite Cells, Skeletal Muscle
Totipotent Stem Cells
Multipotent Stem Cells
Pluripotent Stem Cells
Mesoderm

**Table 2:** Nouns that occur >100 times in *stem cell *MEDLINE references

Ranking	Score	Noun
1	43.8	mesoderm
2	37.7	fibroblast
3	33.6	Stem
4	29.6	foreskin
5	28.0	mesenchyme
6	27.0	progenitor
7	26.3	noggin
8	25.4	epiblast
9	24.8	endoderm
10	23.7	tenon
11	23.5	somite
12	23.3	Zellweger
13	23.0	gastrulation
14	22.7	notochord
15	22.2	ectoderm
16	22.0	XP
17	21.5	ES
18	20.6	xeroderma
19	19.5	Cockayne
20	18.9	myotome
21	18.7	gastrula
22	18.1	CFC
23	17.8	keloid
24	16.0	granulocyte-macrophage
25	15.7	haematopoiesis
26	15.5	blastula
27	15.3	Rous
28	15.0	Werner
29	14.7	BMP
30	14.4	stem

**Table 3:** Adjectives that occur >100 times in *stem cell *MEDLINE references

Ranking	Score	Adjective
1	51.3	embryoid
2	35.7	somital
3	32.4	mesodermal
4	30.5	totipotent
5	27.8	fibroblastic
6	19.4	mesenchymal
7	18.6	semisolid
8	18.5	immortal
9	17.1	haemopoietic
10	15.4	committed
11	14.4	ectodermal
12	14.3	vegetal
13	13.3	hematopoietic
14	13.2	senescent
15	12.2	diploid
16	12.1	dermal
17	12.0	endodermal
18	11.8	skinned
19	11.7	confluent
20	11.3	sonic
21	11.2	haematopoietic
22	10.6	myogenic
23	10.5	erythropoietic
24	10.5	embryonic
25	10.4	quiescent
26	10.4	morphogenetic
27	9.2	inductive
28	9.1	primitive
29	9.1	dorsoventral
30	8.7	unscheduled

**Table 4:** Verbs that occur >100 times in *stem cell *MEDLINE references

Ranking	Score	Verb
1	11.6	immortalize
2	10.9	plait
3	10.6	engraft
4	10.5	skin
5	9.7	purge
6	9.2	seed
7	9.2	subculture
8	8.5	reprogram
9	8.3	rejoin
10	8.2	passage
11	7.6	stem
12	7.4	recapitulate
13	7.2	nucleate
14	7.0	proliferate
15	6.8	culture
16	6.6	condition
17	6.4	transform
18	6.2	ruffle
19	6.1	deregulate
20	6.1	explant
21	5.4	populate
22	5.4	rescue
23	5.3	wound
24	5.2	round
25	5.0	migrate
26	4.9	cultivate
27	4.7	sort
28	4.3	transplant
29	4.5	differentiate
30	4.3	internalize

**Table 5:** High scoring references not annotated with *stem cell *MeSH terms.

Ranking^1^	PMID	Title
139	9811585	Hematopoietic induction and respecification of A-P identity by visceral endoderm signaling in the mouse embryo.
160	8714368	The role of fibroblast growth factor-2 (FGF-2) in hematopoiesis.
174	11672504	Molecular regulation of embryonic hematopoiesis and vascular development: a novel pathway.
177	8805699	Positional cloning of a global regulator of anterior-posterior patterning in mice
404	2910353	Dual role of fibronectin in hematopoietic differentiation.
426	10441547	Regulative development of the sea urchin embryo: signalling cascades and morphogen gradients.
638	11730936	The dynamics of bone marrow stromal cells in the proliferation of multipotent hematopoietic progenitors by substance P: an understanding of the effects of a neurotransmitter on the differentiating hematopoietic stem cell.
720	3659868	Early cardiogenesis in the newt embryo.
750	79573	Calcium-binding protein of the chick chorioallantoic membrane. II. Vitamin K-dependent expression.
801	7538068	A conserved enhancer of the human and murine Hoxa-7 gene specifies the anterior boundary of expression during embryonal development.

^1 ^Rank was assigned by computing the average score of all the nouns present in the abstract and title of an article, and comparing this score with that of other articles in the merged list of 162,832 references. The merged set was constructed from 81,416 references randomly selected from MEDLINE combined with 81,416 references that are annotated with the MeSH term stem cells or one of its children in the MeSH hierarchy.

## References

[B1] NLM (2004). MEDLINE. http://www.ncbi.nlm.nih.gov/PubMed.

[B2] NLM (2004). Medical Subject Headings (MeSH). http://www.nlm.nih.gov/mesh/filelist.html.

[B3] Mitchell TM (1997). Machine Learning.

[B4] Yang Y, Liu X (1999). A re-examination of text categorization methods. Annual ACM Conference on Research and Development in Information Retrieval.

[B5] Kim W, Aronson AR, Wilbur WJ (2001). Automatic MeSH term assignment and quality assessment. Proc AMIA Symp.

[B6] Wilbur WJ (2000). Boosting naive Bayesian learning on a large subset of MEDLINE. Proc AMIA Symp.

[B7] Forman G, Cohen I (2004). Learning from Little: Comparisons of Classifiers Given Little Training: 4 AD/9/20; Pisa, Italy..

[B8] Salton G (1989). Automatic Text Processing -- The Transformation, Analysis, and Retrieval of Information by Computer.

[B9] Andrade MA, Valencia A (1998). Automatic extraction of keywords from scientific text: application to the knowledge domain of protein families. Bioinformatics.

[B10] Andrade MA, Bork P (2000). Automated extraction of information in molecular biology. FEBS Lett.

[B11] Perez-Iratxeta C, Keer HS, Bork P, Andrade MA (2002). Computing fuzzy associations for the analysis of biological literature. Biotechniques.

[B12] Dumais ST, Platt J, Heckerman D, Sahami M (1998). Inductive learning algorithms and representations for text categorization. In CIKM-98: Proceedings of the Seventh International Conference on Information and Knowledge Management.

[B13] Netzel R, Perez-Iratxeta C, Bork P, Andrade MA (2003). The way we write. EMBO Rep.

[B14] Manning C, Schütze H (1999). Foundations of Statistical Natural Language Processing.

[B15] Simpson JA and Weiner ESC (1989). Oxford English Dictionary.

[B16] Harris MA, Clark J, Ireland A, Lomax J, Ashburner M, Foulger R, Eilbeck K, Lewis S, Marshall B, Mungall C, Richter J, Rubin GM, Blake JA, Bult C, Dolan M, Drabkin H, Eppig JT, Hill DP, Ni L, Ringwald M, Balakrishnan R, Cherry JM, Christie KR, Costanzo MC, Dwight SS, Engel S, Fisk DG, Hirschman JE, Hong EL, Nash RS, Sethuraman A, Theesfeld CL, Botstein D, Dolinski K, Feierbach B, Berardini T, Mundodi S, Rhee SY, Apweiler R, Barrell D, Camon E, Dimmer E, Lee V, Chisholm R, Gaudet P, Kibbe W, Kishore R, Schwarz EM, Sternberg P, Gwinn M, Hannick L, Wortman J, Berriman M, Wood V, de la CN, Tonellato P, Jaiswal P, Seigfried T, White R (2004). The Gene Ontology (GO) database and informatics resource. Nucleic Acids Res.

[B17] Hahn U, Romacker M, Schulz S (2002). Creating knowledge repositories from biomedical reports: the MEDSYNDIKATE text mining system. Pac Symp Biocomput.

[B18] Barnes JC (2002). Conceptual biology: a semantic issue and more. Nature.

[B19] Shah PK, Perez-Iratxeta C, Bork P, Andrade MA (2003). Information extraction from full text scientific articles: where are the keywords?. BMC Bioinformatics.

[B20] Wilbur WJ, Yang Y (1996). An analysis of statistical term strength and its use in the indexing and retrieval of molecular biology texts. Comput Biol Med.

[B21] Institut fuer maschinelle Sprachverarbeitung US (2004). Tree-Tagger.

[B22] Perez-Iratxeta C, Bork P, Andrade MA (2001). XplorMed: a tool for exploring MEDLINE abstracts. Trends Biochem Sci.

